# Reasons and Resolutions for Inconsistent Variant Interpretation

**DOI:** 10.1155/2023/4955235

**Published:** 2023-05-22

**Authors:** Liling Lin, Hong Pan, Yu Qi, Yinan Ma, Ling Qiu

**Affiliations:** ^1^Department of Laboratory Medicine, Peking Union Medical College Hospital, No. 1 Shuaifu Yuan, Dongcheng District, Beijing 100730, China; ^2^Department of Central Laboratory, Peking University First Hospital, No. 8, Xishiku Street, Xicheng District, Beijing, China 100034; ^3^State Key Laboratory of Complex Severe and Rare Diseases, Peking Union Medical College Hospital, Peking Union Medical College & Chinese Academy of Medical Sciences, Beijing 100730, China

## Abstract

In the postgenomic era, variant interpretation is crucial for diagnosing monogenic diseases, which is the premise of precision medicine. The bottleneck and difficulty of genetic disease diagnosis have switched from the inaccessibility of detection technology to the interpretation of sequencing results. Multiple studies have suggested that the inconsistency rate of interlaboratory variant interpretation is approximately 10~40%. However, many clinicians have not paid enough attention to this area at present. In this review, we summarized the reasons for inconsistency, including classification methodology, information obtained by the interpreter, evidence application, and expert judgement. For clinicians, genetic counsellors, and molecular pathologists, it is necessary to reevaluate genetic reports, especially those supported by old literature and databases in clinical practice. For unresolvable cases, pedigree analysis, collaboration with research labs for functional experiments, and long-term follow-up to combine advanced clinical presentations with updated data and literature are needed.

## 1. Background

For monogenic diseases, variant interpretation determines the genetic diagnosis, and the latter influences clinical management, prognosis prediction, and prevention. Next-generation sequencing (NGS) includes whole-genome sequencing (WGS), whole-exome sequencing (WES), and disease-focused GS/ES (virtual panel or hybrid capture panel). Compared with the reference sequence, a single WES analysis could reveal ten thousand variants, of which 1055 are novel on average [1]. A single WGS could identify 3.5 million variants, of which an average of 600 thousand are rare or novel [2]. Therefore, NGS has a higher demand for unified and consistent variant interpretation, and deciphering these variants is one of the major bottlenecks in genomic medicine. The inconsistencies in variant interpretation would lead to discrepant genetic diagnoses, impacting the results of clinical studies on the natural history, phenotype-genotype correlation, treatment, and prognosis of genetic diseases.

In 2000 and 2007, the American College of Medical Genetics (ACMG) released recommended standards for the interpretation of sequence variants [3, 4]. As the predecessors to the 2015 guideline, previous guidelines classified the variants into five to six categories (reported and causative, unreported and expected to be causative, unreported and may or may not be causative, unreported and probably not causative, reported and neutral, and unknown variant but associated with a clinical presentation) and briefly described the grading criteria. However, the grading pathway was not explicated clearly. Hence, many clinical genetic laboratories have developed variable in-house interpretation protocols [5–10], causing inconsistent variant classifications and genetic diagnoses [11, 12].

Facing the vast number of variants identified by NGS and the urgent demand for a unified interpretation method, the American College of Medical Genetics and Genomics and the Association for Molecular Pathology (ACMG/AMP) issued an updated guideline in 2015 [13]. The guideline has been widely used in laboratories and by clinicians. As a survey in 2019 showed, in the Gene Test Registry [14], 95% (62/65) of responding laboratories had adopted the guideline.

The guideline, which standardized the framework for Mendelian-disease variant interpretation, is most commonly used in the interpretation of single-nucleotide variants (SNVs) and small indels (20~22 bp or less than 50 bp). Variants are classified into five categories, namely, pathogenic (P), likely pathogenic (LP), variant of uncertain significance (VUS), likely benign (LB), and benign (B). Twenty-eight criteria covering the clinical phenotype, population frequency, literature reports, in silico prediction, and functional experiments were developed and are explained in the guidelines. Interpreters are recommended to combine multidimensional evidence and personal experience to evaluate a variant. However, for extensive adaptability, several words are not very concise in some criteria, such as “extremely low frequency for recessive conditions” in PM2. Laboratories set different thresholds for the allele frequency (AF), which might be 0.1%, 0.5%, or 1%, and thus, differences occur [11].

Therefore, interlaboratory inconsistencies have not been minimalized. In this review, we summarized the reasons and resolutions for interlaboratory inconsistencies from published literature to inspire future directions for practice and research.

## 2. Methodology for Literature Review and Consistency Evaluation

We searched PubMed for studies on the consistency of variant interpretation between Jan 1, 2015, and Aug 1, 2022, using the terms “variant interpretation” AND “consistency OR inconsistency OR comparison OR accordance OR agreement”. Full-text articles using the 2015 ACMG/AMP criteria in Mendelian diseases and their related citations were read and cited. Articles comparing interlaboratory inconsistency rates are summarized in Table 1.

The difference in the classification of variation between laboratories is classified as “five-tier class inconsistency,” “three-tier class inconsistency,” or “differences affecting clinical treatment (medically significant difference, MSD)“ [11]. Five-tier class inconsistency indicates a difference among P, LP, VUS, LB, and B. Three-tier class inconsistency refers to the difference among the three classification levels (P/LP, VUS, and LB/B), and MSD is the difference between P/LP vs. nonactionable variations (VUS, LB, and B). Among inconsistent variants, those that can achieve consistency through reanalysis, data sharing, interlaboratory discussion, etc., are called “resolvable variants;” variants that do not achieve consistency after these efforts are called “unresolvable variants” [15].

## 3. Overview of Inconsistent Rates and Reasons

As shown in Table 1, for a short period of time, after the recommendation was launched, the inconsistency rate based on the five-tier class reached 66%. The rate dropped to 29% through efforts on reanalysis, data sharing, and interlaboratory discussion [11]. In 2020, 5 years after the release of the 2015 ACMG/AMP recommendation, the five-tier inconsistency rate declined to 16%. The rate of three-tier class inconsistency varied from 10 to 40% and declined by 3.5 to 25% after attempts to resolve the disagreement.

In the early stage, the variant classification protocol is one of the most frequent reasons for discrepancies [16]. The appearance of the 2015 edition guideline has largely solved this problem. In addition, differences in relevant information, discordance in criteria application, and personalized expert judgement may have an influence on variant classification. We will discuss these issues in detail in the following content.

## 4. Differences in Relevant Information

Collecting information on AF in population databases and searching disease databases and literature reports are prerequisites for variant classification. Analysis time and the use of different in-house databases and public databases would result in interlaboratory asymmetric information, leading to discrepancies in interpretation. Reciprocal reanalysis and data exchange among laboratories can minimize these kinds of differences. To reduce discrepancy more holistically, AMP released a statement to incentivize public data sharing of variants across clinical laboratories [17].

### 4.1. Phenotype and Phenotype-Genotype Relationship

In both phenotype-driven and nonphenotype-driven analyses, the usage of PP4 (phenotype specific to a known disorder) relies on accurate and comprehensive phenotype curations and phenotype-genotype matching [13]. The discrepancy in information about phenotypes and gene-disease associations may cause interlaboratory inconsistencies in variant interpretation.

Phenotype curations should be performed by experienced clinicians. For some diseases, at the time of genetic testing, symptoms may be atypical or not yet fully manifested and will emerge during follow-up, which requires long-term observation [18]. For example, one of the major difficulties in interpreting fetal exome sequencing lies in the limited clinical presentations at an early stage [19].

Variants in genes with uncertain significance can only be interpreted as VUS. Studies [15] have shown that the stronger the association between a gene and a disease and the better deciphering of the gene function, the lower the proportion of VUS variants and the higher the number of benign or pathogenic variants in the gene. Data on genotype-phenotype associations will accumulate or change over time. Inconsistency may occur when laboratories employ different genotype-phenotype information. Timely updating of related information may help reduce interlaboratory inconsistencies [15]. ClinGen [20] has been updating gene-disease validity standard procedures and scores. GenCC [21] commits to uniform and consolidate gene-disease relationships from multiple databases, comprising ClinGen, OMIM, Orphanet, in-house databases, etc. These efforts facilitate integrating real-time updated phenotype-genotype association information and make it accessible across laboratories.

### 4.2. Analysis Time

The time factor, referring to the time when the assessment took place, has a greater influence on the frequency of evidence. Early studies compared the odds ratio (OR) of the variants in the case group with that in the control group. The control group was generally only a few hundred people in size. After the emergence of large-scale public population databases, such as ESP (2015) [22], 1000G (2015) [23], ExAC (2016, integrated into gnomAD) [24], and gnomAD (2017) [25], many originally “disease-causing” variants that did not exist in small-scale control groups were found to be frequent in large-scale populations; thus, they are now classified as benign variants [12, 26].

Moreover, before the well-accepted 2015 edition guideline, laboratories developed their own standards, causing a higher rate of discrepancy before 2015. A study conducted by Yang et al. [27] showed that the proportion of outliers in the variants uploaded before 2014 in the ClinVar database (3.6%) was higher than that after 2014 (0.5~0.8%).

In 2016, the Emory University Genetics Laboratory reanalysed over 16,000 variants [15] before June 2015. They modified the classification of nearly 6.3% (998/16000) of the variants, and due to updated AF data, 60% (592/998) of the variants changed from VUS to LB/B. A study performed by Harrison et al. [16] showed that after only one laboratory carried out a reanalysis with new data, nearly 50% (112/242) of the variants reached an agreement.

In summary, it is recommended to reanalyse variants using new standardized criteria and evidence rather than cite the results documented in earlier literature or databases directly.

### 4.3. Different In-House Databases

There are differences in internal data of cosegregation, coexistence variant, frequency, and phenotype [16]. In one laboratory, a variant might be found to be inconsistent with cosegregation (BS4), coexist with another pathogenic variant (BP2), or have a high AF in in-house databases (BA1, BS1, or BS2). Differences can occur if another laboratory fails to access these data. One study [16] suggested that data sharing can resolve nearly 30% (70/242) of three-tier inconsistencies.

### 4.4. Different Public Databases

The public databases used by different laboratories, including population databases, in silico forecasting tools, and disease or variant databases, are not completely consistent, which generates inconsistent results. Population databases, in silico forecasting tools, and disease databases are discussed in Sections 4.2, 5.2.3, and 4.1, respectively.

Several criteria, such as PS1, PS2, PS3/BS3, and PM5, require information on previous literature. Due to differences in public databases and search strategies, there are differences in the references cited for the classifications reported by different laboratories. For example, in the study by Gradishar et al. [28], for the same variant, three laboratories cited different references to support their classifications. HGMD, one of the most widely used variant databases, comprehensively curated variants from published literature. While the subscription version provides timely up-to-date information, this information is inaccessible to those who do not subscribe [29], which may cause incomplete and inconsistent information bias. For variants not annotated with high scores in ClinVar, Zhang et al. recommended using variant2literature, LitVar, Mastermind Genomic Search Engine, and Variant Information Search Tool (VIST), which extract variant information from text or images, improving searching efficiency [30].

Different public databases account for approximately 5% (11/242) of inconsistencies [16], and a consensus could be reached through interlaboratory communication.

## 5. Discordance in Evidence Application

Based on the same data, different laboratories might apply different criteria. Criterion misuse, different criterion application protocols, and weight adjustment can result in this kind of discordance.

### 5.1. Misusing or Misunderstanding the Criteria

In 2016, Amendola et al. [11] found that several criteria in the classification guideline were not well clarified, and misuse of the criteria was not rare. For this reason, the Clinical Sequencing Exploratory Research (CSER) consortium added annotation to the items that were easily misunderstood. For example, for frequency evidence (PM2, BS1, and BS2), the sample size of the normal population should be no less than 1,000 individuals or 2,000 alleles. PS1 should only be applied when compared with the known causative variants, and the new missense variants have the same amino acid changes but different nucleotide changes. By 2020, the CSER consortium evaluated the consistency among the same nine laboratories for the second time and found that the rate of evidence misuse has been reduced.

### 5.2. Criterion Application Difference

Under the premise that the grading criteria are understood and applied correctly; interpreters might apply different criteria based on the same data, causing discordance in criterion application. This reflects the subjectivity of the guideline. Several studies [11, 15, 16, 31, 32] have indicated that this kind of difference is one of the most common causes of interlaboratory discrepancy. Only partial agreement can be reached on this kind of discrepancy through communication and discussion.

In a study performed by Amendola et al. [11], 27 out of 28 criteria were applied differently several times. The most frequent was PP4 (the patient's phenotype/family history is highly consistent with the known disease); in contrast, PVS1 (loss of function variant) had the least discordance. The main controversies regarding the criterion application and ClinGen solutions are listed in Table 2.

#### 5.2.1. Phenotypic Evidence

The controversy over phenotypic evidence is attributed to the judgement of whether it is “highly consistent” with known phenotypes of a disease (PP4). In Harrison et al.'s research [16], 10% (3/31) of MSD variants had different PP4 applications. In the specific guidelines issued by ClinGen-SVI for some disease-causing genes, the clinical and laboratory presentations for diagnosing the disease are clarified, but most diseases still lack specific explanations.

#### 5.2.2. Frequency Evidence

Frequency criteria, including PM2, PS4, BS1, BS2, and BA1, are common for application differences [11, 15, 16, 31, 32]. The reason is that various laboratories set different AF threshold values for these frequency criteria. A study conducted by Garber et al. in 2016 showed that [15] the differences due to different AF thresholds caused most of the five-tier discordance, such as P vs. LP and LB vs. B, which seldom impacted clinical management. A study published by Harrison et al. in 2017 [16] showed that 45% of the variants that still have differences after reanalysis, and discussion had differences in frequency evidence applications. Additionally, the difference in the use of BS1 (AF greater than expected for the disorder) and BS2 (observed in a healthy adult) was the main cause of MSDs.

#### 5.2.3. Computational Prediction Evidence

PP3 and BP4, which are predicted to be pathogenic or benign by multiple in silico tools, are not well clarified in the guideline. Since various computational tools have been developed to calculate the potential pathogenicity of missense or splicing variants, different laboratories might have inconsistent results when using different algorithms and standards. Missense variants are an example of this. Among 16 kinds of prediction software, MutationTaster [33] (protein structure/function and evolutionary conservation), PolyPhen-2 [34] (protein structure/function and evolutionary conservation), SIFT [35] (conservation), and PROVEAN [36] (homology) are preferred by many domestic laboratories. Some laboratories would use three scoring tools for prediction and apply PP3 when the results are all harmful [37]. Some laboratories would use four structure or function-predicted tools plus one conservation algorithm. PP3 is applied when the variant is highly conservative and predicted to be harmful by more than 2 tools [38]. Some studies used 6 tools and considered PP3 when 4 types of software predict harmfulness [39]. Some studies used 12 types of predictive software without describing criteria for giving PP3/BP4 [40]. Nevertheless, other studies have used the metaprediction software REVEL [41].

In the gene-specific guidelines issued by the ClinGen-SVI working group, needled recommendations for different genes are presented. For example, in the deafness gene guideline [41], the recommended prediction software for missense variants is REVEL. It is specified that PP3 should be used when the REVEL score is >0.7 (or 0.75) and that BP4 should be used when the REVEL score is <0.15. In addition, the recommended splicing prediction software is MaxEntScan. While in the *CDH1* gene guideline [42], no missense prediction software is recommended. It is mentioned that for splicing variants, PP3 could be applied when at least three of the four splicing prediction software tools are splicing-affected. The listed splicing prediction software includes Human Splicing Finder (HSF), Maximum Entropy (MaxEnt), Berkeley Drosophila Genome Project (BDGP), and ESEfinder.

Li et al. [43] studied the diagnostic performance of 23 missense prediction software programs and found that, generally, the tools with the best performance were REVEL [44], VEST, and the combination of the two methods, ReVe [43]. However, the gold standard in this study was the opinions uploaded by most laboratories on ClinVar instead of functional experiments.

Although the application of computational evidence varies widely among laboratories, this type of evidence has a small impact on variant classification [45]. In 2017, Harrison et al.'s [16] research showed that 16% of unresolved variants have discordance in computational prediction evidence (PP3, BP4, and PM5); only 2 MSD variants out of 31 variants that did not reach agreement on three-tier classification exhibited inconsistent PP3/BP4 application.

#### 5.2.4. Functional Evidence

According to the 2015 ACMG/AMP guideline, functional evidence included PS3/BS3 (supported by well-established functional experiments), PM1 (variants located in the variant hot spot/functional domain), and PP2 (missense variants on genes for which missense variants are often pathogenic and less benign). Harrison et al. [16] found functional evidence accounting for 48% of inconsistent variants, all of which were MSDs (P/LP vs. VUS/LB/B). A study performed by Amendola et al. in 2020 [31] showed that PM1 was one of the three most controversial criteria.

## 6. Professional Judgement and Weight Adjustment Rationale

The differences originating from professional judgement involve low-penetrance variants, variant types, special genes, and weight adjustment rationale.

### 6.1. Low-Penetrance Variants

Low-penetrance variants are controversial points [27]. One condition is when a variant of high population AF (>1%) is frequently found in cis with another pathogenic variant in autosomal dominant inherited disease, and the patient presents disease-specific phenotypes. Another condition is called “pseudodeficiency” [15]. In autosomal recessive disease, the homozygous type of the variant only causes a mild or no phenotype. In this case, the enzyme activity is reduced but does not reach the defective level. When this variant is in trans with another pathogenic variant, forming compound heterozygosity, the phenotypes are concordant with the disease, and the enzyme activity is below the defective level. Some interpreters would define these two conditions as benign evidence, while others may classify them into low-penetrance variants. These variants have potential pathogenicity in some patients and might enhance the harm of pathogenic variants.

Low-penetrance variants may also be called “modifiers,” “risk alleles,” “susceptible variants,” and “variants relative to the disease but not disease-causing.” Some laboratories tend to classify these variants as benign. In Furqan et al.'s study [32], among the 11 unanimous variants, nearly two-thirds of the variants were classified as “modified variants” by at least one laboratory. Such variants accounted for a high proportion of inconsistent variants in the ClinVar database [27]. Frequency evidence, coexistence evidence, and literature evidence of these variants are prone to inconsistencies among laboratories. Specific guidelines are needed to standardize the nomenclature and classification standards of such variants [16].

### 6.2. Types of Variants

Several studies have discovered that discordance rates vary depending on the variant type. Garber et al. found that nearly 25% (72/293) of the total inconsistent variants and 43% (72/166) of the unresolved inconsistent variants among laboratories were synonymous (silent) or intronic variants, which might be due to a difference in the underlying philosophy on classification [15]. Some laboratories tended to classify such variants as benign variants, while others classified them as VUS unless there were significant data to support benign or pathogenic classification. Although it appeared unresolvable, the discordance on benign and VUS had little impact on clinical management [15].

While in Yang et al.'s research [27], among the variants uploaded by multiple submitters in ClinVar, the concordance rates of silent variants, truncating variants, protein sequence changes (mostly missense variants), and splicing variants were 99.8, 98.8, 94.7, and 97.5%, respectively. They concluded that missense variant classification is the most daunting challenge.

### 6.3. Special Genes

Variants in some special genes have a high rate of inconsistency among laboratories. A study [15] reported that variants in *TTN* (MIM: 188840) genes accounted for approximately 18.5% (30/166) of the unresolved inconsistent variants. *TTN*, which encodes titin, is a causative gene of dilated cardiomyopathy. The AF of truncated variants of *TTN* in the population is higher than the expected prevalence. Hence, the ACMG criteria are not applicable. Each laboratory has formulated its own *TTN* gene interpretation protocol, leading to a high inconsistency rate.

### 6.4. Weight Adjustment Rationale

The guideline [13] indicates that the weight of criteria could be adjusted based on expert judgement and evidence collected, which gives rise to inconsistencies in the weight adjustment procedure. To better quantify the weight and harmonize the procedure, ClinGen-SVI released several general recommendations on classification rationale (https://clinicalgenome.org/working-groups/sequence-variant-interpretation/). Weight adjustment is often related to frequency criteria (PM2, PS1), cosegregation criteria (PP1), coexisting variant criteria (PM3, BP2), functional experiment criteria (PS3), and PS1 (same amino acid changes as known pathogenic variants) [11].

A growing number of gene-/disease-specific guidelines are also available on the ClinGen website (https://cspec.genome.network/cspec/ui/svi/). The gene-/disease-specific guidelines made adjustments to the phenotype, frequency, functional experiment, and cosegregation criteria, which are determined based on the characterization of the gene or disease. The refinements are summarized in Table 2.

## 7. Resolution of Inharmonious Variant Interpretation

Methods for improving the consistency of variant classification include the use of standardized training guidelines, reevaluation/reanalysis of intralaboratory variants, data sharing and academic discussion between laboratories, and developing gene-/disease-specific guidelines.

### 7.1. Standardized Training in Interpretation

Two CSER studies [11, 31] conducted at a four-year interval indicated that training on the use of guidelines can reduce errors in the misuse of classified criteria and increase consistency. As ClinGen-SVI is committed to releasing updates and supplements to the guidelines from time to time, the content of the training should evolve progressively.

### 7.2. Reanalysis and Reevaluation

As mentioned above, data on a variant and its related gene will accumulate and change over time, and these revisions could have a profound impact on classification. Therefore, regular or timely data reanalysis should be emphasized. In 2019, ACMG issued a statement recommending that each laboratory should establish a workflow of variant reassessment/reanalysis [18], clarifying indications for reassessment/reanalysis, procedures, intervals, whether to change reports, whether to notify patients, and related costs. Reevaluation refers to the reevaluation of key variants based on the latest clinical data, and reanalysis refers to the reanalysis of the original NGS data.

The periodicity of reanalysis and reevaluation is not explicated in the statement, and clinical laboratories are encouraged to have separate policies on these issues [18]. The ClinGen-VCEPs declared that they would reevaluate all LP or VUS variants at least every 2 years [30]. Considering the rapid progress of molecular genetics, some experts have proposed that the interval should be shorter. The suggested reevaluation/reanalysis indications are as follows: (1) when there is a request from the outside, including the laboratory, clinician, or patient;(2) when a variant of the previous classification is found in a new patient or newly reported study; (3) when the guidelines or population data are updated; and (4) before important clinical decisions. The analysis cycle may vary based on different types of variants. LP and VUS variants are more prone to upgrade or downgrade than P, LB, and B variants and should be reevaluated more frequently [18].

### 7.3. Data Sharing and Communication

The method of data sharing includes sharing in-house databases among laboratories and uploading variants to public databases. The ClinVar [46, 47] database comprises curated sequence variants uploaded by global laboratories and research institutions. Initially, only a few users were anticipated to upload the criteria for classification [27]. To achieve the desired effect, ClinVar encouraged uploaders to share the criteria and evidence in detail instead of simple results. Both the number of variants and the consistency rate of variant classification among laboratories have been increasing every year on the ClinVar website.

### 7.4. Gene-/Disease Specification Criteria

The ClinGen-SVI working group [20] is committed to improving the consistency of sequence variant interpretation and has issued disease-/gene-specification criteria (https://cspec.genome.network/cspec/ui/svi/). To date, specification criteria for 14 types of diseases, 62 nuclear genes, and mtDNA (last search time on Aug 8th, 2022) have been released. The major improvements involve AF thresholds, functional domains, clinical diagnostic criteria, and recommended in silico tools. The overwhelming majority of genes have no gene-specific guidelines, and thus, there is much to be done in this area. New inconsistencies arise due to whether the laboratories will update the internal protocol in time based on the specification criteria [31]. Therefore, more efforts are needed to fully achieve this goal.

### 7.5. Exploring Gene Functions and Genotype-Phenotype Correlations

For the inconsistent variants that originated from expert judgement and limited or controversial knowledge and could not reach a consensus after data sharing and reevaluation, more studies on pathogenesis, pedigree analysis, and natural history based on accumulating cases to elucidate gene functions and genotype-phenotype correlations are needed.

## 8. Conclusion

While NGS technology benefits the diagnosis of monogenic diseases, inconsistencies in the classification of sequence variants remain challenging. Factors that impact variant interpretation comprise classification methodology, information obtained by the interpreter, evidence application, and expert judgement. For clinicians, genetic counsellors, and molecular pathologists in clinical practice, it is necessary to reevaluate genetic reports, especially those supported by old literature and databases. For unresolvable cases, pedigree analysis, collaboration with research labs for functional experiments, and long-term follow-up to combine advanced clinical presentations with updated data and literature are needed.

We appeal that, in the future, variant reevaluation and reanalysis should be routinely carried out as histopathological consultations in clinical laboratories. More efforts should be made to establish a strategy for reevaluation and analysis, which would be more feasible with funding support.

## Figures and Tables

**Table 1:** Summary of interlaboratory discrepancy rates reported in the literature.

No.	Article	Number of variants	Source of variants	Enrichment time	Disease types	Inconsistent rate			
						Five-tier	Three-tier	MSDs	VUS vs. LB/B
1	Amendola et al., 2016 [11]	99	9 CSER labs	Before Dec 2015	Unlimited	66%→29%	41%→14%	22%→5%	19%→9%
2	Garber et al., 2016 [15]	293	EGL and 4 labs on ClinVar	Before Jun 2015	Neuromuscular disorders, skeletal dysplasia, and short stature	56.7% (163/293)	33% (98/293)	N.M.	N.M.
3	Furqan et al., 2017 [32]	112	SHaRe	Before Mar 2015	Hypertrophic cardiomyopathy	N.M.	20.5%→10.7%	17%→9.8%	N.M.
		695	ClinVar			N.M.	45.20%	N.M.	N.M.
4	Harrison et al., 2017 [16]	6169	4 labs on ClinVar	Before Jan 2016	Unlimited	N.M.	11.7%→8.3%	5%→2%	N.M.
5	Yang et al., 2017 [27]	27224	ClinVar	Before Oct 2016	Unlimited	N.M.	^∗^MC: 89.3% CC: 81%	^∗^MC: 96.7% CC: 94.1%	N.M.
6	Harrison et al., 2018 [48]	49242	686 submitters on ClinVar	Before Apr 2017	Unlimited	N.M.	22.20%	4.30%	N.M.
		24445	41 clinical labs		Unlimited	N.M.	15.40%	2.70%	N.M.
7	Amendola et al., 2020 [31]	158	8 CSER labs	Before Aug 2020	59 genes reported by accidental discovery	16%	29.8%→10.8%	11%→4.4%	N.M.

Five-tier inconsistent rate: P vs. LP vs. VUS vs. LB vs. B. Three-tier inconsistent rate: P/LP vs. VUS vs. LB/B. MSDs, medically significant difference: P/LP vs. VUS/LB/B. The number marked by “^∗^” is the consistent rate. CSER: Clinical Sequencing Exploratory Research consortium, EGL: Emory Genetics Laboratories, SHaRe: Sarcomeric Human Cardiomyopathy Registry, N.M.: not mention, CC: complete consensus, MC: major consensus. If a variant is graded by 3 or more laboratories, the degree of agreement can be divided into CC and MC [27]. CC means that all laboratories have the same classification results, while MC indicates that the majority have the same classification results. The definition of “majority” is that the agreed laboratories account for two-thirds or more of all laboratories.

**Table 2:** Criterion application differences in variant classification and modified criteria proposed by CLIGEN.

Type of evidence	Original classification criteria	Descriptions that may cause the discrepancy	Modified criteria
Phenotypic evidence	PP4: the phenotype or family history is highly specific and consistent with a single-gene disorder.	Phenotype curation impacts the application. Judgement of high specification and consistency may vary [18].	Some gene-specific guidelines provided well-defined diagnostic criteria for PP4 [49].

Frequency evidence	PS4: the prevalence of the variants in the affected population is significantly higher than that in the control population.	Not applicable to rare variants [13].	1. ClinGen-SVI suggested to downgrade PM2 (extremely low AF) to PM2_Supporting. It was recommended to refine the classification rules to adapt to changes in PM2 (for example, PVS1+PM2_Supporting should be classified as LP), but lack of clear quantitative standard. 2. ClinGen-SVI released an exception list on variants with AF > 0.05 but having potential significance. 3. Some gene-specific guidelines provided recommended precise thresholds for PM2/BA1/BS1/BS2 [41].
	PM2: absent or at an extremely low frequency in population databases.	Laboratories may use different population databases [32]. The AF thresholds may vary among laboratories [11, 39, 40, 50, 51].	
	BA1: AF is >5% in the population databases.		
	BS1: AF is greater than disease prevalence.		
	BS2: in fully penetrant diseases, the variants were found in healthy adults as homozygosity for AR, heterozygosity for AD, or hemizygosity for XL inheritance.	The judgement on disease penetrance may be inconsistent [11].	

Computer/predictive evidence	PP3: multiple in silico algorithms predict the variant to be deleterious.	Labs may have different strategies for multiple tools [15]. Using different computer prediction software can lead to discordance [11, 15].	1. ClinGen-SVI launched calibration of computational tools for missense variant pathogenicity classification [52]. 2. Several gene-specific guidelines provided recommended in silico tools.
	BP4: multiple in silico algorithms predict the variant to be benign.		
	BP7: a synonymous variant would not impact splicing and is predicted by splicing prediction algorithms.		

Functional experimental evidence	PS3: well-established in vitro or in vivo functional studies show damaging effects on genes and gene products.	The definition of “well-established” functional experiments may vary among laboratories [11].	ClinGen-SVI proposed a decision-making workflow for evaluating PS3/BS3 [53].
	BS3: well-established in vitro or in vivo functional studies show no damaging effects.		

Literature/database reports	PP5: reputable reports consider the variant pathogenic.	Definition of “reputable source” may not be uniform and the references or databases may vary.	ClinGen-SVI suggested to discontinue the use of these two criteria.
	BP6: reputable reports consider the variant to be benign.		

Null variant	PVS1: a null variant in a disease-causing gene where the loss of function (LOF) is a deleterious mechanism.	Laboratories may have divergence in judging whether LOF is the genetic pathogenic mechanism of the gene [54]. Sometimes, variants nearing the C-terminal may impact the protein function [55].	ClinGen-SVI proposed a detailed decision-making process for PVS1 refinement based on variant type, pathogenic mechanism, variant location, and inherited pattern [54].

Missense variants/in-frame insertions or deletions	PS1: a novel variant has the same amino acid alteration as a known pathogenic variant.	The use of different databases for searches for “identified pathogenic variants” can lead to differences in results.	1. Defining weather PP2/BP1/PM1 are applicable in some gene-specific guidelines [49]. 2. The ClinGen evidence repository released expert-curated variants for applying PS1/PM5 efficiently.
	PM5: a novel missense variant leading to a novel amino acid change at the same locus as a known pathogenic variant.		
	PP2: a missense variant in a gene where these types are often pathogenic.	The specific ranges and thresholds for the frequency words “common,” “low rate,” and “primarily” are not clear enough. For example, “major” can range from “most (>50%)” to “all” or “almost all (>90%)” [11].	
	BP1: a missense variant in a gene where pathogenic variants are primarily truncating.		
	PM1: a variant in a mutational hot spot region and well-established functional domain.	Discordance in the “mutational hot spot” and “functionally defined domains without benign variants” [11].	

Cosegregation evidence	PP1: cosegregation with the disease in multiple affected family members (stronger evidence if more evidence).	It is not clear how to adjust the strength of evidence for cosegregation.	1. CSER proposed the rule on cosegregation evidence weight adjustment based on inheritance pattern and a number of affected or unaffected family members [56]. 2. Well-defined in some gene-specific criteria [41].
	BS4: lack of segregation in affected family members.		

Coexisting evidence	PM3: a variant detected in trans with a pathogenic variant in a recessive disorder.	The criteria are not suitable for “low penetrance variants.” Judgement of low penetrant variant is highly variable.	1. ClinGen-SVI proposed a point-based system to determine the strength of in trans observations (ACMG/AMP criterion PM3). 2. Well-defined in some gene-specific guidelines [49].
	BP2: a variant found in cis with a pathogenic variant in the AD gene with full penetrance or a known pathogenic variant on the same chromosome in any pattern of inheritance.		

Weight adjustment	The level of evidence may be appropriately adjusted based on the evidence collected and professional judgement.	It is not clearly specified when, how, and to what extent the adjustment will be made [15].	ClinGen-SVI proposed refined nomenclature of criterion weight adjustment, such as PP1_Moderate/strong. For PS2/PM6 (de novo evidence) and PM3 (in trans criterion), ClinGen-SVI proposed scoring systems for weight adjustment.

AF: allele frequency; SVI: the Sequence Variant Interpretation Working Group; CSER: Clinical Sequencing Exploratory Research consortium.
